# The “impossible” rectal anastomosis: a novel use for endoluminal vacuum-assisted therapy

**DOI:** 10.1007/s10151-020-02363-x

**Published:** 2020-11-20

**Authors:** Nadja C. Lehwald-Tywuschik, Andrea Alexander, Nour Alkhanji, Georg Flügen, Stephen Fung, Alexander Rehders, Wolfram Trudo Knoefel

**Affiliations:** Department of Surgery, University Hospital Duesseldorf and Heinrich-Heine-University Duesseldorf, Moorenstrasse 5, 40225 Duesseldorf, Germany

**Keywords:** Vacuum therapy, Prophylaxis, Anastomosis, Endosponge

## Abstract

**Purpose:**

Low rectal anastomoses can safely be performed, usually secured by a diverting ostomy. However, in cases of inflammation, extensive scarring, after extensive radiation, or after severe stapler dysfunction the risk for an anastomotic leak may become prohibitively high. We present a novel use for endoluminal vacuum-assisted therapy (EVAT) for otherwise “impossible” low rectal anastomoses.

**Methods:**

Our initial series consisted of 14 consecutive patients who underwent prophylactic EVAT treatment due to unsafe low colorectal anastomosis. The vacuum sponge was placed intraoperatively in cases otherwise calling for a Hartmann’s procedure. An open-pored polyurethane sponge was placed prophylactically transanally for a mean duration of 11 days. Patient characteristics, complications, and risk factors were prospectively collected from medical records and analyzed.

**Results:**

Between March 2017 and September 2019, we performed this novel technique in 14 patients enabling us to perform an anastomosis. Our collective consisted of 4 female (29%) and 10 male (71%) patients with a medium age of 59 years. Underlying disease was colorectal cancer in 10 patients, ovarian cancer, perforated sigmoid diverticulitis, ischemic colitis and sarcoma in one patient each. Dominant factors putting the anastomosis at extremely high risk were acute inflammation (*n* = 2), frozen pelvis (*n* = 2), intraoperative local chemotherapy (*n* = 2), stapler dysfunction (*n* = 2), non-closable rectal stump (*n* = 2), empty pelvis (*n* = 1) and ultra-low anastomosis (*n* = 3). Prophylactic EVAT was successful in 92% and gastrointestinal continuity was preserved in all patients.

**Conclusion:**

This is the first description of prophylactic EVAT treatment. It seems to be a simple and safe method to enforce the high-risk low rectal anastomosis.

## Introduction

Anastomotic leakage is a major complication in colorectal surgery and the main cause for postoperative morbidity and mortality. Despite advancement of surgical technique and perioperative management in the past decade, leakage rates of colorectal anastomoses were reported between 8 and 20% even in the presence of a diverting stoma [1]. If risk factors such as neoadjuvant radiotherapy, inflammation or low level of the anastomosis are present, the risk for colorectal anastomotic leakage is significantly increased. In low anastomoses (< 3 cm from the anal verge), the risk is even sixfold higher [2].

For many years, vacuum-assisted closure (VAC) therapy has been widely used for septic wound closure or the treatment of abdominal fascia dehiscence [3]. Vacuum therapy with negative pressure via a vacuum-sealed sponge resulted in faster healing due to enhanced tissue granulation, drainage of infected wounds, increased vascularization and decreased bacterial colonization. In recent years, the endoluminal vacuum-assisted therapy (EVAT) was invented to resolve presacral abscess cavities and to treat anastomotic leakages after colorectal surgery [4]. It was first described by Weidenhagen et al. in 2008, who have used EVAT for anastomotic leaks after anterior rectal resection [5]. Since then, multiple retrospective studies have reported the effect of EVAT for anastomotic leakages with healing rates up to 70% [6].

To the best of our knowledge, we here present a novel indication of EVAT to prevent anastomotic leakage of high-risk anastomoses in colorectal surgery, which to date has never been described. The aim of this study was to evaluate the use of prophylactic EVAT in 14 patients who received a high-risk anastomosis. This novel method seems to be beneficial to protect high-risk low colorectal anastomoses.

## Materials and methods

### Patients

Between 03/2017 and 10/2019, all patients who underwent colorectal surgery with a low rectal anastomosis at the Department of Surgery, Heinrich-Heine-University Hospital Duesseldorf, Germany were evaluated for the risk to develop postoperative problems involving the anastomosis by one of the senior colorectal surgeons (A.R., W.T.K.). Fourteen patients who received prophylactic endoluminal vacuum therapy due to an unsafe rectal anastomosis were included in this study. All collected data were adhered to the guidelines established by the Declaration of Helsinki and has been approved by the local ethics committee (2020–840).

Clinical data were collected from patients’ medical records, compiled into an Excel®-file database, and analyzed. The following data were collected: demographic parameters including age, gender, diagnosis, risk factors, surgical characteristics including type of procedure, reason for endosponge therapy, as well as complication rate.

### Endoluminal vacuum-assisted (EVAT) therapy

The present study only includes patients with prophylactic EVAT to secure the low anastomosis without any signs of anastomotic leakage. All 14 patients have received EVAT due to a high-risk rectal anastomosis.

The endosponge was placed intraoperatively after the anastomosis was completed.

**Fig. 1:**
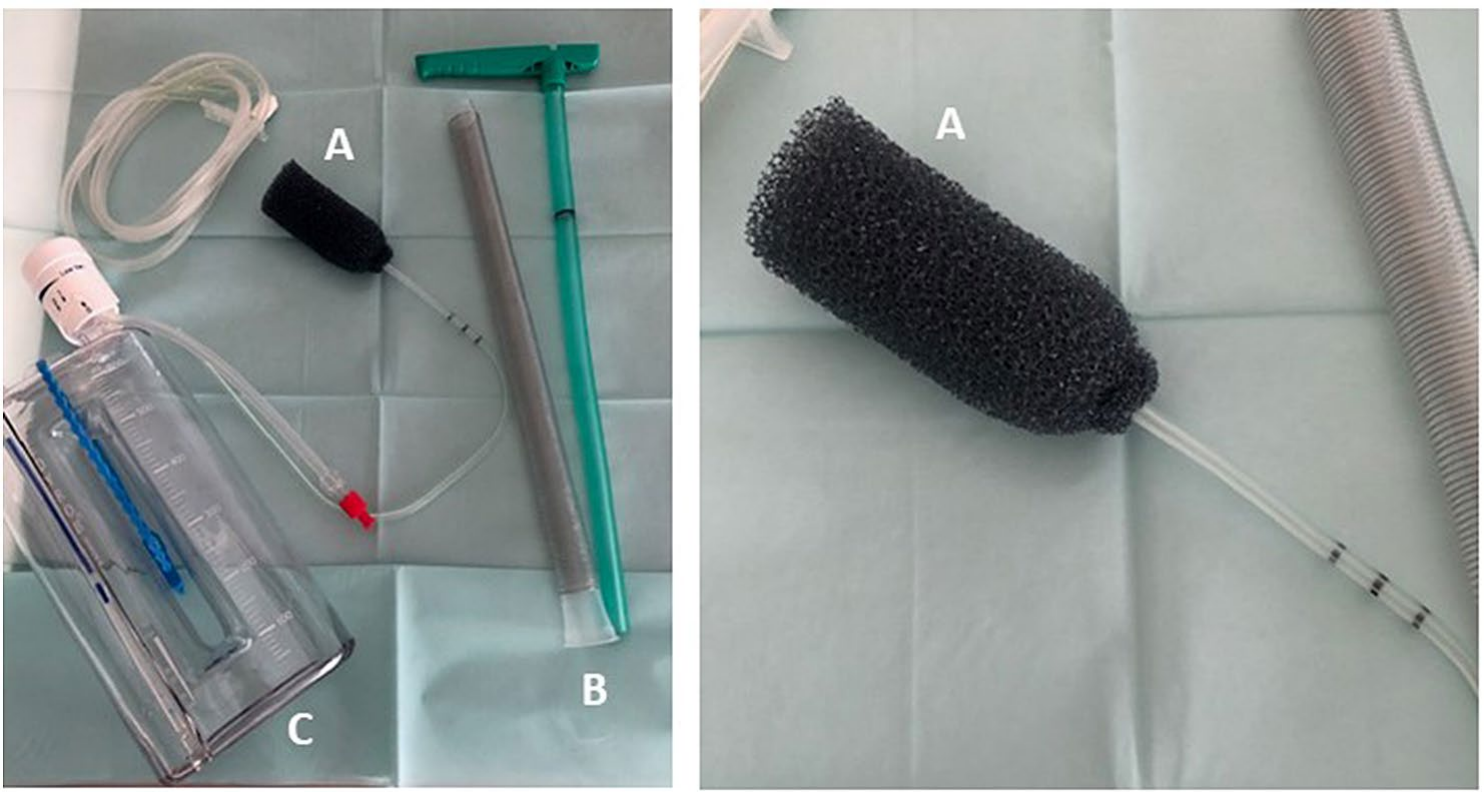
Endosponge introduction system: **a** open-cell polyurethane foam endosponge (B. Braun Medical BV, Melsungen, Germany), **b** introducer tube, **c** fluid collection bottle with constant negative pressure (Redyrob® TRANS PLUS suction device, Melsungen, Germany)

Endoluminal vacuum-assisted therapy is a technique in which an open-cell polyurethane foam sponge (B. Braun Medical BV, Melsungen, Germany) is positioned transanally (Fig. 1a). The placement is done intraoperatively under general anesthesia. The sponge is cut according to the size and diameter of the rectal anastomosis (Fig. 1a). First, the anastomosis is palpated by digital-rectal examination. An endoscope was not used since all anastomoses were easily reached by digital examination. The sponge is placed alongside the finger or into the introducer tube (Fig. 1b) and then inserted transanally next to the palpating finger for positioning control. In the latter case, the sponge is then pushed out of the tube and placed at the level of the anastomosis. The introducer tube is then carefully removed. Correct positioning of the sponge at the anastomosis is controlled by digital-rectal examination. The finger is then carefully removed leaving the sponge in place. The endosponge is then connected to a bottle with constant negative pressure (Redyrob® TRANS PLUS suction device, Melsungen, Germany) (Fig. 1c). Due to the tonus of the anal sphincter, no other dressing or tape is needed to secure the negative-pressure system. The sponge system needs to be changed every 3–6 days to prevent adherence of the mucosa. This can be done without sedation or analgesia at the bedside depending on the patients’ preference.

### Statistical analysis

Statistical analysis and graphing were performed using MS Excel. All results are expressed as median with range or mean ± standard deviation. As the number of patients included in this study was small, no formal comparisons of subgroups were performed.

## Results

Between 03/2017 and 10/2019, this novel technique of prophylactic EVAT was applied on 14 cases enabling a safe anastomosis in high-risk patients after colorectal surgery.

Our patient collective (*n* = 14) consisted of ten (71%) male and four (29%) female patients (Table 1). Median age was 59 years (range 36–79 years). The mean body mass index in our collective was 26.3.

**Table 1:** Patient characteristics

Patient characteristics	*n* = 14
Age years (mean ± SD)	59.1 ± 11.8
Sex *n* (%)	
Male	10 (71.4)
Female	4 (28.6)
BMI kg/m^2^ (mean ± SD)	26.3 ± 5.7
Diagnosis *n* (%)	
Colorectal cancer	10 (71.4)
Ovarian Cancer	1 (7.1)
Sarcoma	1 (7.1)
Perforated sigma diverticulitis	1 (7.1)
Ischemic colitis	1 (7.1)
Neoadjuvant therapy *n* (%)	
Yes	5 (35.7)
No	9 (64.3)
Loop ileostomy *n* (%)	14 (100)
Surgical procedure *n* (%)	
LAR	9 (64.3)
Colectomy	4 (28.6)
RHP	1 (7.1)
Anastomosis level cm (mean ± SD)	4.7 ± 1.6
Stapled anastomosis *n* (%)	14 (100)
End to end	11 (78.6)
Ileoanal pouch	3 (21.4)
Loop ileostomy *n* (%)	14 (100)

*BMI *Body mass index,* LAR *Low anterior resection,* RHP *Reversal of Hartmann’s procedure

Ten patients underwent surgery for colorectal cancer (71%) (Table 1). Further indications for the remaining four patients were: ovarian cancer (*n* = 1; 7%), sarcoma (*n* = 1; 7%), perforated sigmoid diverticulitis (*n* = 1; 7%) or ischemic colitis (*n* = 1; 7%). Five patients of the 12 oncologic patients have been treated with neoadjuvant radiotherapy (36%).

In all patients, open surgery was performed. Nine patients (64%) underwent low anterior resection. Colectomy was performed in 4 cases (29%) and one patient underwent reversal of a Hartmann’s procedure (7%) (Table 1).

The mean distance of the anastomosis from the anal verge was 2.8 cm (range 1.6–4.9 cm) (Table 1). All patients received a stapled anastomosis. 11 patients received an anastomosis in an end-to-end technique, whereas an ileoanal pouch was created in three patients. A diverting stoma was constructed in all 14 patients to secure the high-risk colorectal anastomosis.

In all 14 patients, EVAT was started during the initial surgery and was placed under general anesthesia. Further endoluminal sponge exchanges were done at bedside or in the operating room under light sedation or analgesia.

**Fig. 2:**
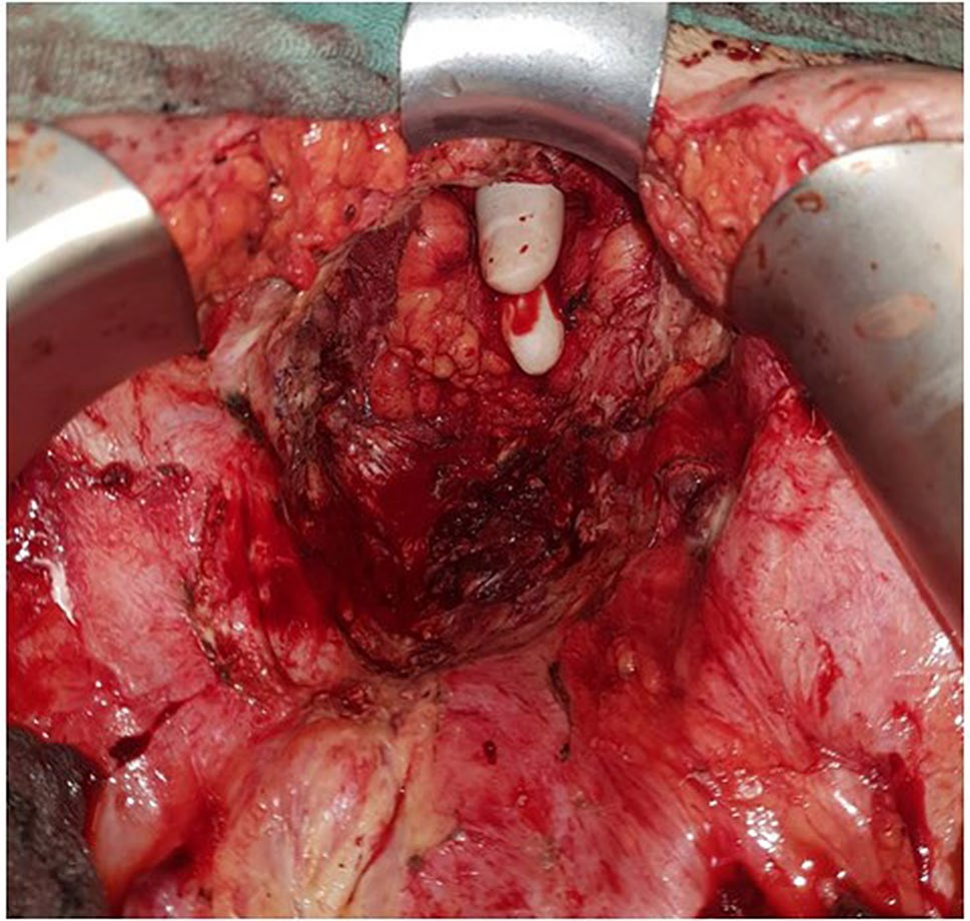
A difficult intraoperative situation—a female pelvis, after radiation and exenteration and non-closable, short rectal stump

The indication for prophylactic EVAT therapy was at the discretion of the senior colorectal surgeon based on various risk factors of the low colorectal anastomosis: acute inflammation (*n* = 4), frozen pelvis (*n* = 4) or empty pelvis (*n* = 3) (Table 2; Fig. 2). In two patients, a stapler dysfunction was observed. Two patients had a non-closable rectal stump. Hyperthermic intraperitoneal chemotherapy (HIPEC) with empty pelvis was applied in two patients, which was the indication for prophylactic endoluminal sponge placement (Table 2). Without EVAT, the responsible surgeon would have considered a Hartmann procedure to be the standard of care in all patients to minimize the risk for the patient.

**Table 2:** Characteristics of endosponge therapy

	*n* = 14
EVAT	
Reason for EVAT *n*	
Acute inflammation	4
Frozen pelvis	4
Stapler dysfunction	2
Empty pelvis	3
Non-closable rectal stump	2
Intraoperative local chemotherapy	2
Endosponge treatment days (mean ± SD)	10.9 ± 7.7
No. of endosponge changes *n* (%)	2.1 ± 1.8
Morbidity *n* (%)	
Yes	1 (7.1)
No	13 (92.3)
Type of morbidity *n* (%)	
Anastomotic leakage	1 (7.1)
Anal bleeding	1 (7.1)
Follow-up	
Duration of stoma before closure days (median + SD)	188.7 ± 114.9
Stoma closure *n* (%)	12 (85.1)
Stenosis of the anastomosis *n* (%)	1 (7.1)

*EVAT *endoluminal vacuum-assisted therapy, *No *number

In our collective, EVAT lasted for a median of 11 days (range 2–28 days). The device was replaced every 3–5 days for a median of 2.1 (range 0–6) times (Table 2).

Prophylactic EVAT was successful in 92% (*n* = 13) (Table 2). One patient experienced complications (7%) during EVAT. In this patient, the sponge exchange was complicated by an anal bleeding caused by mechanic traction due to delayed sponge change (after 7 days). In this case, we also observed a coloanal anastomotic leakage which was treated with extended endovac treatment for 28 days. However, the anastomotic leak was successfully treated and gastrointestinal continuity was preserved. In all other patients, healing of the anastomosis occurred without complications or leak.

The stoma was preserved for a mean of 188.7 days (range 2–365 days) before it was closed. Closure of the stoma was done is 12 patients (85.1%). Two patients were lost to follow-up (14.8%). Both came from foreign countries and follow-up was presumably performed where they lived. A stenosis of the anastomosis was observed in one patient (7.1%) which was treated by repeated endoscopic dilatation before the stoma was successfully reversed after 365 days.

## Discussion

Prophylactic endoluminal vacuum-assisted treatment can prevent anastomotic leakage in high-risk anastomoses after colorectal surgery in this first clinical experience.

The aim of our novel approach was to perform a safe colorectal anastomosis and to avoid Hartmann’s procedures under challenging conditions. Gastrointestinal continuity could be sustained in all of our patients. Despite one anastomotic leakage, healing of the anastomosis succeeded in all patients.

Since all the reconstructions were high-risk anastomoses, we constructed a diverting stoma in all patients. It has already been described that EVAT is less effective without the presence of a diverting stoma [2, 6]. Patients with EVAT for anastomotic leakage demonstrate a faster recovery in the presence of a stoma [6].

We have also experienced that coloanal anastomoses are less suitable for EVAT because the sponge easily slipped out of the anus verge and no effective vacuum was maintained.

Since we have applied the vacuum treatment intraoperatively for prophylaxis, only a short intervention time with a median time of 11 days was necessary. In case of therapeutic use for a leakage, the median time of EVAT has been reported to be 13–40 days [4].

The success of vacuum-assisted treatment for anastomotic leakage is known to be positively correlated with early onset of therapy. It has been previously described that early administration of vacuum therapy is an independent predictive factor for the healing rate [2]. In 2009, Koperen et al. described a 75% successful closure rate when vacuum-assisted treatment started within 6 weeks [7]. This finding was further supported by another recent study [8]. Recently, the largest report on endorectal vacuum treatment for leaks reported an improved success rate of 72.4% vs. 27.8% when vacuum therapy was initiated within 15 days after the diagnosis of anastomotic leakage [9].

These encouraging results after early treatment led us to the assumption that prophylaxis may even be superior to early treatment. We, therefore, speculated that immediate administration of vacuum therapy would positively affect the healing of the high-risk anastomoses that are otherwise “impossible” or very risky. Future randomized studies are needed to proof this effect.

Successful healing rates between 56 and 97% of the anastomotic leakage have been reported with vacuum-assisted therapy [5]. The majority described a healing rate > 70%. However, when risk factors for impaired healing are present, the rate was significantly decreased. The major risk factors for failure of the vacuum-assisted treatment were late start for EVAT, neoadjuvant radiotherapy, lack of stoma and age > 60 years [2, 5, 8].

In the CLEAN study, EVAT resulted in a 20% increase of preserved anastomoses and 27% less chronic presacral sinus [8]. Another study by Povanov et al. even reported a 85% success rate of “saved” anastomoses [2].

Complications related to EVAT have been reported in up to 19% [2] and described as bleeding, persistent presacral residual fistula, abscess, sponge migration into the abdomen, anastomotic ulcer or anastomosis stenosis [2, 4]. In line with these findings, we observed one bleeding in the anastomosis region of the coloanal anastomosis caused by the endosponge with a consecutive leak.

Also, the arterial blood supply to the sphincter is stronger than to a short rectal stump and vacuum therapy may promote bleeding. With this experience, we postulate that coloanal anastomoses are more problematic to treat by EVAT. Due to the difficult circumstances concerning the coloanal anastomosis, we experienced a 7% complication rate, which is still lower than described [2].

Since 2007 vacuum-assisted therapy is also routinely applied for treatment of upper gastrointestinal (GI) defects of different etiology after major gastroesophageal surgery [10]. Reported success rates ranged from 84 to 100%. The encouraging endoluminal vacuum-assisted therapy in upper GI leakages has reduced the need of surgical revisions. In a prophylactic setting, EVAT is currently evaluated after esophagectomy and might be a beneficial tool for high-risk upper GI anastomosis. Yet, the risk for stenosis and dysphagia is substantial in our own experience for this novel approach.

The major limitation of our study is the low number of patients and missing comparative collective. There is a selection bias because of subjective evaluation of the anastomosis and the decision for treatment at the discretion of the senior colorectal surgeon. Although the novel prophylactic EVAT approach seems to safely improve the postoperative outcome of the very high-risk colorectal anastomosis, this technique needs to be validated in a larger series of patients in a controlled prospective trial. Furthermore, future studies should evaluate which patients benefit from prophylactic vacuum-assisted therapy and determine long-term outcome. Since this is a safe method, EVAT might also be beneficial for low-risk anastomoses.

## Conclusion

To date, our clinical experience with prophylactic endoluminal vacuum-assisted therapy for high-risk anastomoses is limited; however, this initial study presents a novel use of prophylactic EVAT as a promising low-risk approach in extremely difficult cases of colorectal surgery to perform a colorectal anastomosis to restore gastrointestinal continuity.

## Data Availability

The datasets used and/or analyzed during the current study are available from the corresponding author on reasonable request.

## Abbreviations

EVAT: Endoluminal vacuum-assisted therapy
GI: Gastrointestinal
HIPEC: Hyperthermic intraperitoneal chemotherapy
ICU: Intensive care unit
VAC: Vacuum-assisted closure
